# Prevention of hypothermia in patients undergoing orthotopic liver transplantation using the humigard® open surgery humidification system: a prospective randomized pilot and feasibility clinical trial

**DOI:** 10.1186/s12893-017-0208-z

**Published:** 2017-01-23

**Authors:** Laurence Weinberg, Andrew Huang, Daniel Alban, Robert Jones, David Story, Larry McNicol, Brett Pearce

**Affiliations:** 1 Department of Surgery, and Anaesthesia Perioperative and Pain Medicine Unit, The University of Melbourne, Melbourne, Australia; 2 Department of Anaesthesia, Austin Hospital, Heidelberg, Australia; 3 Liver and Intestinal Transplant Unit, Austin Hospital and The University of Melbourne, Heidelberg, Australia; 4 Perioperative and Pain Medicine Unit; The University of Melbourne, Victoria, Australia

**Keywords:** Temperature, Anaesthesia, Nasopharyngeal, Thermoregulation, Liver, Transplant

## Abstract

**Background:**

Perioperative thermal disturbances during orthotopic liver transplantation (OLT) are common. We hypothesized that in patients undergoing OLT the use of a humidified high flow CO_2_ warming system maintains higher intraoperative temperatures when compared to standardized multimodal strategies to maintain thermoregulatory homeostasis.

**Methods:**

We performed a randomized pilot study in adult patients undergoing primary OLT. Participants were randomized to receive either open wound humidification with a high flow CO_2_ warming system in addition to standard care (Humidification group) or to standard care alone (Control group). The primary end point was nasopharyngeal core temperature measured 5 min immediately prior to reperfusion of the donor liver (Stage 3 − 5 min). Secondary endpoints included intraoperative PaCO_2_, minute ventilation and the use of vasoconstrictors.

**Results:**

Eleven patients were randomized to each group. Both groups were similar for age, body mass index, MELD, SOFA and APACHE II scores, baseline temperature, and duration of surgery. Immediately prior to reperfusion (Stage 3 − 5 min) the mean (SD) core temperature was higher in the Humidification Group compared to the Control Group: 36.0 °C (0.13) vs. 35.4 °C (0.22), *p* = 0.028. Repeated measured ANOVA showed that core temperatures over time during the stages of the transplant were higher in the Humidification Group compared to the Control Group (*p* < 0.0001). There were no significant differences in the ETCO_2_, PaCO_2_, minute ventilation, or inotropic support.

**Conclusion:**

The humidified high flow CO_2_ warming system was superior to standardized multimodal strategies in maintaining normothermia in patients undergoing OLT. Use of the device was feasible and did not interfere with any aspects of surgery. A larger study is needed to investigate if the improved thermoregulation observed is associated with improved patient outcomes.

**Trial registration:**

ACTRN12616001631493. Retrospectively registered 25 November 2016.

## Background

The Victorian Liver Transplantation Unit at Austin Health provides liver transplantation services to people in Victoria, Tasmania and parts of Southern New South Wales, Australia. Over 1000 liver transplants have been performed since 1988. Local data from our service shows that during orthotopic liver transplantation (OLT) over 70% of recipients are hypothermic (core temperature less than 36 °C prior to reperfusion of the donor liver), despite standardized measures employed to maintain temperature homeostasis. Hypotherrmia during OLT can result in cardiac arrhythmias and ischaemia, coagulopathy, increased allogeneic transfusion, wound infection delayed post-anaesthetic recovery, shivering and patient discomfort, and prolonged hospitalization. During OLT, hypothermia results from the combination of a large and open surgical wound, prolonged exposure of abdominal organs to room air, compounded by blood loss, massive transfusion, and the need for large volumes of intravenous replacement. Further, being a highly metabolically active organ, removal of the native liver further diminishes overall heat production. Replacement with a cold donor liver preserved in ice introduces an additional hypothermic insult upon revascularization, and the use of extracorporeal circuits (for veno-venous bypass or renal replacement therapy) can further compound thermal stress.

**Fig. 1:**
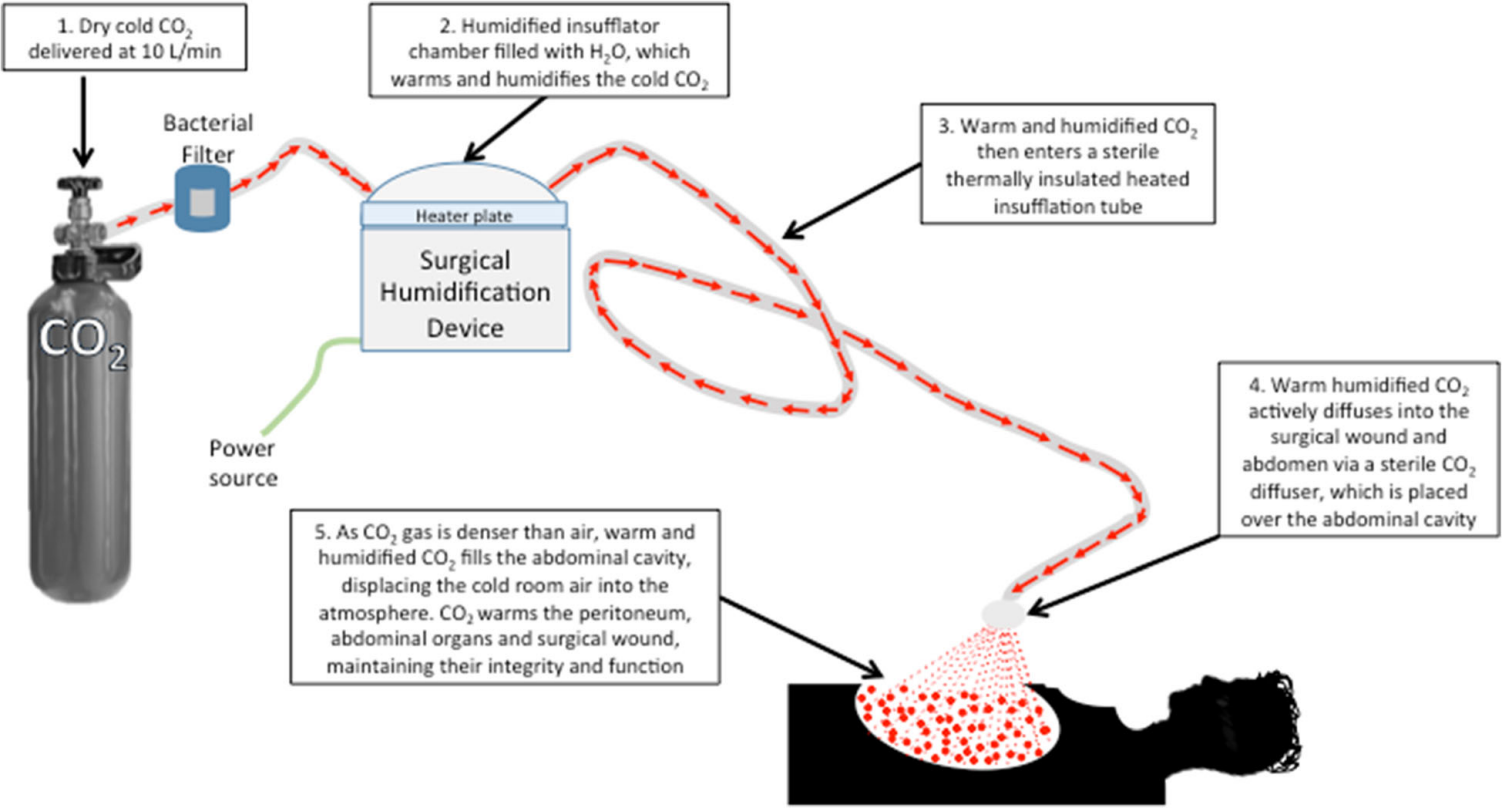
Schematic representation of the Humigard® surgical humidification system

The Humigard® system (Fisher and Paykel Healthcare, Auckland, New Zealand) is a heat delivery system allowing insufflation of carbon dioxide (CO_2_) into the surgical wound. A schematic overview of the humidification system is presented in Fig. 1. Using this system wound ventilation with warmed, humidified CO_2_ has been shown to reduce hypothermia in laparoscopic [1–3], colonic [4, 5] and cardiac surgery [6, 7], however there are no studies evaluating its use in OLT. Therefore, we hypothesized that in patients undergoing OLT the use of the Fisher & Paykel Humigard® system maintains higher intraoperative temperatures when compared to standardized multimodal strategies to maintain thermoregulatory homeostasis.

## Methods

The study was approved by the Austin Health Research Ethics Committee (HREC no: 2012/04674), and was conducted between July 2013 to July 2014 at a university teaching hospital with expertise in liver transplantation. We retrospectively registered the trial with the Australian New Zealand Clinical Trials Registry: ACTRN12616001631493 (registered 25 November 2016). Inclusion criteria included adult recipients (age >18 years) undergoing primary OLT. Exclusion criteria included pregnancy, fulminant hepatic failure, redo-OLT, requirement for continuous veno-venous bypass, haemofiltration, and multi-visceral transplantation. All patients were evaluated preoperatively at a dedicated anaesthesia pre-admission clinic and provided written informed consent.

### Primary end point

The primary end point was core temperature measured 5 min prior to reperfusion of the donor liver (Stage 3 − 5 min). Measurement of core temperature was measured using a nasopharyngeal temperature probe (CareFusion Incorporation, Australia) inserted in the upper third of the nasopharynx [8]. Nasopharyngeal temperature has been reported to be an accurate and precise measurement of core body temperature [9–11]. As the insertion of a continuous Cardiac Output Swan-Ganz pulmonary artery catheter (Edward Lifesciences CCO-Combo, IL, USA) is part of our standard anaesthesia technique for all patients undergoing OLT, we also used temperature measurements from the PAC. The PAC is considered as the gold standard measurement of core body temperature [12–14].

### Secondary end points

Secondary end points included temperature measurements at the following time points:

- Stage 1 + 60 min: (recorded 60 min after start of the dissection phase)
- Stage 2 + 30 min (recorded 30 min after start of the anhepatic phase)
- Stage 3 + 5 min: (recorded 5 min post reperfusion)
- Stage 3 + 60 min: (recorded 60 min post reperfusion)
- Closure of the surgical wound

Other data collected included baseline patients characteristics, baseline temperature (recorded at surgical incision), indication for transplant, MELD, SOFA and APACHE II scores, use of vasoactive drugs and inotropes, fluid intervention, transfusion requirements, estimated blood loss, minute ventilation and PaCO_2_ (measured at the same temperature time points) and duration of surgery.

### Standardization of perioperative temperature homeostasis

Perioperative temperature homeostasis was standardized for all participants. One hour prior to surgery, participants were pre-warmed with a full body warming blanket (Bair Hugger 3 M™, Model 315) set at 43 °C. On arrival to the operating room, the ambient operating room temperature was set at 21 °C, and participants were placed on sterile full access underbody warming blanket (Bair Hugger 3 M™, Model 637) set at 43 °C, placed over a standard operating table. These warming blanket devices were continued during induction of anaesthesia and during insertion of all invasive monitoring lines, after which the full body warming blanket was replaced with an intraoperative upper body warming device that covered both upper limbs and face (Bair Hugger 3 M™, Model 523XL) for the remainder of the case. During the anhepatic phase, ambient operating temperature was increased to 23 °C, with no further adjustments for the remainder of the case. Intraoperatively, crystalloid and colloid fluid intervention, including the use of packed red blood cells were delivered via a Belmont® Rapid Infuser RI-2 system that delivered preheated fluids at 42 °C. If clinically indicated, fresh frozen plasma, platelet and cryoprecipitate transfusions were delivered via a separate HOTLINE® (Smiths Medical, Kent, UK) intravenous warming fluid device, set at 42 °C.

### Standardization of anaesthesia

Anaesthesia management followed a protocol designed to standardize patient care. This included insertion of two arterial lines, central venous and Continuous Cardiac Output Swan-Ganz pulmonary artery catheters (Edwards Lifesciences, Vigilance II, CCO- Combo, IL, USA) used in conjunction with the Vigilance II monitor (Edwards Lifesciences, IL, USA). Three liver transplant anesthesiologists and three dedicated transplant surgeons provided anaesthesia and surgical care to all participants. For all participants, general anaesthesia was induced with fentanyl (3–5 ug/kg), propofol (1–2 mg/kg), and cisatracurium (0.2 mg/kg). Maintenance of anaesthesia included inhalation of isoflurane in a 50% oxygen:air mixture. Mechanical ventilation was standardized and a circle circuit with absorption of CO_2_ at 1 L/minute fresh gas flow was used. End-tidal CO_2_ was maintained at 35–45 mmHg. Noradrenaline was administered at the discretion of the anaesthetists maintaining the mean arterial pressure within 20% of the patients’ preoperative values. Fluid intervention included the acetate buffered crystalloid solution, Plasmalyte-148^TM^ (BaxterHealthcare, Toongabie, NSW, Australia), and Albumex 20% (CSL, Biotherapy, Victoria, Australia). Blood and other blood products were administered at the discretion of the anaesthetists and based on conventional laboratory coagulation tests, in addition to point of care thromboelastography.

As part of our intuition’s OLT surgical protocol, all patients had a Makuuchi’s incision (reverse L- Incision) for surgical exposure to allow dissection and hepatectomy of the native liver. Immediately after surgical excision and opening of the abdomen, all patients had the Humigard system deployed into the right cranial quadrant of the abdomen by the surgical team. The diffuser system was attached as per device instructions. Participants randomized to the Humigard system had CO_2_ delivered into the abdominal cavity at 37 °C and 100% relative humidity. Participants randomized to the control group did not receive delivery of CO_2._ Removal of the native liver began with dissection of the hilum carried down to the hepatic artery. Anhepatic phase commenced when the portal vein was transected. After the removal of the native liver, the allograft implantation began with the suturing of the donor upper vena cava to the hepatic veins preserving flow through the inferior vena cava using a partial side clamp (the ‘piggy-back’ technique). After the portal and arterial anastomoses were completed, the biliary tract reconstruction was completed using an end-to-end choledocostomy without a T-tube stent. In select cases, due to inadequacy of the biliary duct size, or depending on the underlying disease, a Roux-en-Y hepatojejunostomy was performed. Prior to surgical closure the Humigard system was removed from the abdomen in all participants.

### Statistics

Samples size calculations were performed using inferences for means comparing two independent samples [15]. This was established with an internal audit of 60 anaesthesia charts of patients undergoing OLT in our institution over the previous 2 years, which demonstrated a mean (standard deviation) temperature prior to reperfusion of 35.4 °C (0.8). Nominating a clinically important difference of 1 °C in the intervention group, using a two-sided test, with an alpha value of 0.05 and a desired power of 0.80, the sample size required for each group was 11 participants. A computer generated randomization program was used to ensure all that participants received individual randomization codes. Random permutations of treatments for each participant were created using the randomization program first generator application entering “Humigard Group” and “Control Group” as the treatment labels. Participant randomization was sealed in an opaque envelope. Study investigators, anaesthesiologists and surgeons were blinded to the intervention. Randomization was performed by a dedicated liver theatre technician at the commencement of surgery who either turned the Humigard device on, or left the device off. Treatment allocation was revealed after data analysis was performed. All clinicians involved in postoperative patient care were also blinded to the intervention.

Continuous data was tested for normality using the D’Agnostino-Pearson omnibus test. For the primary end point between groups, comparisons for continuous data were performed with the Students’s *t*-test. All test were considered two-tailed and a *p*-value <0.05 indicated statistical significance. Values were reported as mean and standard deviation (SD) or medians and interquartile range (IQR). Changes in NPP and PAC temperatures over the given timepoints were measured with repeated measures ANOVA. Given the exploratory nature of this study, no formal adjustment for multiplicity of testing was undertaken, and *p* = 0.05 was regarded as significant for every outcome. This may yield a potential increase in Type 1 error rate, which is acceptable given the pilot and feasibility nature of the study. Statistical analyses were performed using GraphPad Prism version 6.0 (GraphPad Software, La Jolla California). The study is reported according to the updated CONSORT guidelines for reporting parallel group randomized trials [16].

## Results

Twenty-six participants consented for this study. Four participants did not proceed to transplantation, as the donor organ was considered unsuitable. In total 22 participants were randomized, 11 to the Humigard Group and 11 to the Control Group. All participants had the Humigard system positioned without complication. There were no violations in the study protocol, and all participants received the interventions as per allocation. No patients were excluded, and all results have been included in the final analysis. Both groups were evenly matched for baseline characteristics, indications for OLT and intraoperative factors (Table 1). Immediately prior to reperfusion (Stage 3 − 5 min) (primary end point), mean (SD) core temperature was higher in the Humigard Group compared to the Control Group: NPP: 36.0 °C (0.13) vs. 35.4 °C (0.22), *p* = 0.028; PAC: 35.9 °C (0.16) vs. 35.5 °C (0.24), *p* = 0.14). Temperature at each stage of the surgical procedure are summarized in Table 2. There were no significant differences in the ETCO_2_, PaCO_2_, minute ventilation, or inotropic support (Table 2). The median noradrenaline infusion rate prior to reperfusion (Stage 3 − 5 min) was 5 ug/min (IQR 0:9) mcg/min in the Control group and 4 mcg/min (IQR 1:10) in the Humigard group (*p* = 0.96) After reperfusion of the donor liver (Stage 3 + 5 min), mean (SD) NPP temperature decreased to 34.7 °C (0.23) in the Humigard Group and to 35.9 °C (0.22) in the Control Group, *p* = 0.41. Similarly at this time point PAC temperature decreased to 34.9 °C (0.21) in the Humigard Group and to 35.1 °C (0.28) in the Control group, *p* = 0.46. Core temperatures continued to increase in both groups post reperfusion and after 60 min (Stage 3 + 60 min), temperature was higher in the Humigard Group compared to the Control group: Humigard Group NPP: 35.8 °C (0.16) vs. Control Group NPP 35.7 °C (0.15), *p* = 0.77; Humigard Group PAC: 35.8 °C (0.18) vs. Control Group 35.7 °C (0.16), *p* = 0.59.

**Table 1:** Baseline and intraoperative patient characteristics

	Control group (*n* = 11)	Humigard group (*n* = 11)	*P* value
Baseline Characteristics			
Male gender	8 (72%)	7 (63%)	0.72
Age (years)	47.3 ± 13.0	48.0 ± 11.0	0.88
Weight (kg)	83.8 ± 24.1	76.5 ± 15.3	0.40
Body mass index (kg/m^2^)	27.8 ± 7.2	27 ± 4.3	0.61
Indications for transplantation			
Hepatitis C	3	2	
Non-alcoholic steato-hepatitis	1	1	
Autoimmune hepatitis	0	3	
Primary sclerosing cholangitis	2	0	
Primary biliary cirrhosis	1	1	
Hepatocellular cancer	1	3	
Alcoholic cirrhosis	1	0	
Other	2	1	
Prognostic Values			
MELD	19.2 ± 6.4	20.6 ± 8.6	0.66
SOFA	10.1 ± 2.6	11.1 ± 2.2	0.34
Apache II	19.9 ± 4.5	18.4 ± 5.5	0.53
Intraoperative Factors			
Acetate-buffered crystalloid (ml)	3454.6 ± 2841	3681.8 ± 2003	0.39
Albumex (20%) (ml)	436.4 ± 478	490.9 ± 561	0.80
Platelets (pooled adult units)	0 (0:2)	0 (0:1)	0.85
FFP (units)	2 (0:4)	1 (0:3)	0.67
Cryoprecipitate (units)	0 (0:8)	0 (0:9)	0.43
Red Blood cells (units)	0 (0:3)	2 (0:3)	0.43
Donor blood administered (ml)	287.3 ± 149.5	750 ± 240	0.12
Cell saved blood returned (ml)	2319.8 ± 2377	2731.5 ± 3221	0.74
Duration of surgery (hours)	9.7 ± 1.5	9.3 ± 2.3	0.63

Data presented as mean and standard deviation or median and interquartile range

**Table 2:** Temperatures, vasoactive support, End-tidal CO_2_ and minute ventilation

Stage		Control Group (*n* = 11)	Humigard Group (*n* = 11)	Difference	Confidence interval (95%)	*p*-value
Baseline	Ambient Temp (°C)	21.1 ± 0.1	21.1 ± 0.1	0.01 ± 0.2	−0.1 to 0.3	0.95
	PAC Temp (°C)	35.9 ± 0.1	35.8 ± 0.2	−0.1 ± 0.2	−0.6 to 0.4	0.73
	NPP Temp (°C)	35.9 ± 0.1	36.0 ± 0.1	0.1 ± 0.2	−0.4 to 0.6	0.75
	Norad (ug/min)	0 (0,0)	0 (0,0)	-	-	0.99
	PaCO_2_ (mmHg)	37.6 ± 03.59	38.4 ± 4.50	−0.8 ± 1.9	−4.4 to 2.8	0.65
	ETCO_2_ (mmHg)	32.0 ± 2.4	30.9 ± 1.8	−1.1 ± 3.0	−7.4 to 5.2	0.72
	Min Vent (L/min)	5.5 ± 0.2	5.7 ± 0.4	0.20 ± 0.4	−0.7 to 1.1	0.64
1 + 60 min	Ambient Temp (°C)	21.2 ± 0.1	21.3 ± 0.1	0.1 ± 0.2	−0.3 to 0.4	0.73
	PAC Temp(°C)	35.8 ± 0.2	35.7 ± 0.2	−0.1 ± 0.3	−0.7 to 0.5	0.69
	NPP Temp (°C)	35.8 ± 0.2	35.8 ± 0.2	−0.0 ± 0.3	−0.5 to 0.5	0.91
	Norad (ug/min)	1 (0,2)	0.5 (0,2)	-	-	0.90
	PaCO_2_ (mmHg)	37.9 ± 4.2	37.4 ± 6.8	0.5 ± 2.5	−4.5 to 5.5	0.83
	ETCO_2_ (mmHg)	32.1 ± 1.8	31.0 ± 1.3	−1.1 ± 2.2	−5.7 to 3.5	0.62
	Min Vent (L/min)	5.9 ± 0.3	5.7 ± 0.5	−0.2 ± 0.6	−1.3 to 1.0	0.73
2 + 30 min	Ambient Temp (°C)	22.6 ± 0.4	22.5 ± 0.3	−0.12 ± 0.5	−1.1 to 0.8	0.80
	PAC Temp (°C)	35.6 ± 0.3	36.2 ± 0.2	0.60 ± 0.3	−0.1 to 1.3	0.09
	NPP Temp (°C)	35.6 ± 0.23	36.4 ± 0.2	0.78 ± 0.3	0.2 to 1.4	**0.02**
	Norad (ug/min)	5 (0,9)	1 (0,7)	-	-	0.23
	PaCO_2_ (mmHg)	33.0 ± 3.5	36.1 ± 5.8	−3.1 ± 2.1	−7.4 to 1.2	0.15
	ETCO_2_ (mmHg)	27.8 ± 1.2	30.1 ± 1.3	2.32 ± 1.8	−1.3 to 6.0	0.20
	Min Vent (L/min)	6.3 ± 0.3	6.3 ± 0.5	0.01 ± 0.6	−1.2 to 1.2	0.98
3 − 5 min	Ambient Temp (°C)	22.5 ± 0.53	22.6 ± 0.39	0.1 ± 0.7	−1.3 to 1.5	0.89
	PAC Temp (°C)	35.5 ± 0.2	35.9 ± 0.16	0.4 ± 0.3	−0.2 to 1.0	0.14
	NPP Temp (°C)	35.4 ± 0.2	36.0 ± 0.13	0.6 ± 0.3	0.1 to 1.2	**0.03**
	Norad (ug/min)	5 (0,9)	4 (1,10)	-	-	0.96
	PaCO_2_ (mmHg)	33.5 ± 2.9	36.4 ± 5.5	−2.9 ± 2.0	−6.8 to 1.1	0.14
	ETCO_2_ (mmHg)	28.0 ± 1.2	28.9 ± 1.08	1.1 ± 1.6	−2.2 to 4.5	0.48
	Min Vent (L/min)	6.3 ± 0.3	6.4 ± 0.48	0.0 ± 0.6	−1.2 to 1.3	0.93
3 + 5 min	Ambient Temp (°C)	22.5 ± 0.5	22.6 ± 0.5	0.5 ± 0.8	−1.3 to 1.4	0.89
	PAC Temp (°C)	35.1 ± 0.28	34.9 ± 0.21	−0.3 ± 0.4	−1.0 to 0.5	0.46
	NPP Temp (°C)	35.0 ± 0.22	34.7 ± 0.23	−0.3 ± 0.3	−0.9 to 0.4	0.41
	Norad (ug/min)	8 (5,15)	10 (6,15)	-	-	0.76
	PaCO_2_ (mmHg)	35.9 ± 3.9	38.3 ± 4.5	−2.4 ± 1.9	−6.2 to 1.4	0.20
	ETCO_2_ (mmHg)	30.1 ± 1.10	29.5 ± 1.00	−0.6 ± 1.5	−3.7 to 2.5	0.67
	Min Vent (L/min)	6.3 ± 0.34	7.3 ± 0.42	1.0 ± 0.6	−0.2 to 2.1	0.08
3 + 60 min	Ambient Temp (°C	23.0 ± 0.6	22.9 ± 0.5	−0.1 ± 0.76	−1.7 to 1.5	0.88
	PAC Temp (°C)	35.7 ± 0.2	35.8 ± 0.2	0.1 ± 0.24	−0.4 to 0.6	0.59
	NPP Temp (°C)	35.7 ± 0.2	35.8 ± 0.2	0.1 ± 0.22	−0.4 to 0.5	0.77
	Norad (ug/min)	5 (3,10)	6 (5,9)	-	-	0.52
	PaCO_2_ (mmHg)	35.2 ± 4.2	36.4 ± 4.0	−1.2 ± 1.9	−4.9 to 2.4	0.49
	ETCO_2_ (mmHg)	31.0 ± 1.9	29.9 ± 1.3	−1.1 ± 1.72	−4.7 to 2.5	0.53
	Min Vent (L/min)	6.7 ± 0.4	7.14 ± 0.4	0.4 ± 0.57	−0.8 to 1.6	0.46
Closure	Ambient Temp (°C	23.2 ± 0.6	22.8 ± 0.4	−0.4 ± 0.7	−1.8 to 1.0	0.56
	PAC Temp (°C)	36.3 ± 0.1	36.8 ± 0.2	0.5 ± 0.2	−0.1 to 0.1	0.09
	NPP Temp (°C)	36.1 ± 0.1	36.7 ± 0.2	0.5 ± 0.2	0.0 to 1.0	**0.04**
	Norad (ug/min)	4 (1,7)	4 (3,6)	-	-	0.80
	PaCO_2_ (mmHg)	35.6 ± 1.7	34.6 ± 2.9	0.9 ± 2.1	−1.2 to 3.1	0.37
	ETCO_2_ (mmHg)	31.3 ± 1.2	29.0 ± 1.1	−2.3 ± 1.6	−5.6 to 1.1	0.17
	Min Vent (L/min)	7.2 ± 0.5	7.2 ± 0.3	0.0 ± 0.6	−1.2 to 1.2	0.97

Data presented as mean (standard deviation) or median (interquartile range)

*Temp* temperature, *PAC* pulmonary artery catheter, *NPP* nasopharyngeal probe, *Norad* noradrenalin, *ET CO*_*2*_ end tidal carbon dioxide, *Min Vent* minute ventilation

**Fig. 2:**
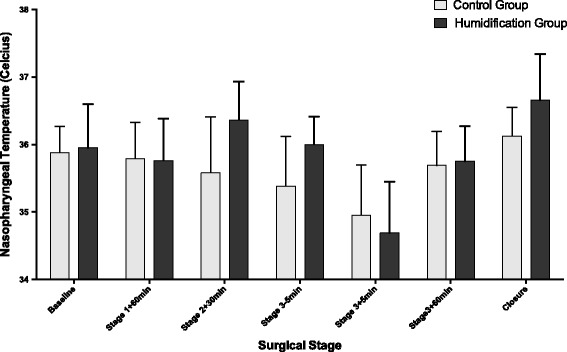
Changes in nasopharyngeal temperature during orthotopic liver transplantation. Data is presented as mean and standard deviation

**Fig. 3:**
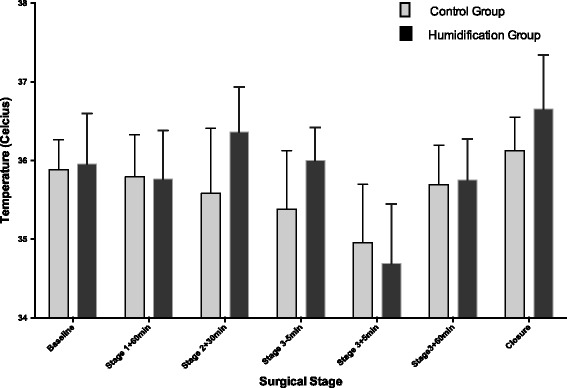
Changes in pulmonary artery pressure temperature during orthotopic liver transplantation. Data is presented as mean and standard deviation

At closure of the abdomen, mean (SD) temperatures were higher in the Humigard group compared to the Control group NPP: 36.7 °C (0.21) vs. 36.1 °C (0.13), *p* = 0.0.045; PAC: 36.8 °C (0.23) vs. 36.3 °C (0.09), *p* = 0.091). At closure, the median noradrenaline requirements were 4 mcg/min (IQR 3:6) in the Humigard Group and 4 mcg/min (IQR 1:7) in the Control Group (*p* = 0.8). There were no differences observed in time on the ventilator, vasoactive support in the ICU, duration of stay in ICU, or length of hospital stay (Table 3). Repeated measured ANOVA showed that both NPP and PAC temperatures over time were higher in the Humigard Group compared to the Control Group (*p* < 0.0001) (Figs. 2 and 3).

**Table 3:** Intensive care and hospital ventilator times, inotropic support and length of stay

	Control Group (*n* = 11)	Humigard Group (*n* = 11)	Difference	Confidence interval (95%)	*p*-value
ICU Ventilation time (hours)	28.9 ± 5.9	30.2 ± 5.3	1.3 ± 7.9	15.4 to 17.7	0.87
ICU Noradrenalin support (hours)	12.3 ± 3.2	30.3 ± 21.6	17.9 ± 21.8	−27.6 to 63.4	0.42
ICU length of stay (hours)	60.9 ± 25.4	89.7 ± 26.9	28.8 ± 37.0	−48.4 to 105.9	0.44
Hospital length of stay (days)	13 (10,15)	13 (10,16)	-	-	0.96

Data presented as mean and standard deviation, or median and interquartile range

## Discussion

We conducted a randomized controlled pilot and feasibility study in patients undergoing liver transplantation to evaluate whether the use of the Fisher & Paykel Humigard® system was superior to standardized multimodal strategies in maintaining normothermia in patients undergoing OLT. Patients in the Humigard Group had higher NPP and PAC temperatures immediately prior to reperfusion (Stage 3 − 5 min) and at skin closure. Importantly, use of the device did not interfere with any aspects of the surgery with 100% adherence and compliance to the study protocol.

Our study demonstrates that core temperature can be increased in patients undergoing OLT by insufflating the wound cavity with humidified CO_2_ via a gas diffuser. There may be several mechanisms underlying this. Firstly, the near 100% relative humidification of the wound cavity substantially reduces heat loss by evaporation. Secondly, as this CO_2_ is warmed to 37 °C, not only are convective losses greatly reduced, but there is a net gain of heat by convection because the humidified CO_2_ is warmer than the wound edges. Moreover, the greenhouse properties of CO_2,_ that is, its ability to absorb and reemit certain wavelengths, reduce heat loss by radiation effectively insulating the patient by providing a thermal blanket [17]. The Humigard system has also been developed to fill an open surgical wound cavity with a laminar flow of CO_2_ for the prevention of arterial air embolism in open-heart surgery [18–21]. However, the localized warming effect of CO_2_ also prevents hypothermia-induced vasoconstriction, which in turn increases tissue oxygen tension of the wound [6]. As CO_2_ has a greater density than air when insufflated with a continuous laminar flow into the wound cavity it gravitates into the wound flooding the cavity, exerting a thermal insulating effect, minimizing the redistribution of heat from the body core to the wound surface, and shielding the patient from diffusion and convective air currents caused by the operating room ventilation [6, 17]. This provides a stable environment of warm humid CO_2_, which increases the efficiency of these mechanisms. Given that the surgical incision during OLT is large and the operation is prolonged, we would also expect these changes to have a more pronounced effect in this group of patients.

Results of our study are consistent with the publication by Frey and colleagues [4], who conducted a randomized controlled trial in patients undergoing open colonic surgery. Frey et al. showed a significant improvement of approximately 0.6 °C in mean core temperature when the Humigard was used compared to controls [4]. Our paper demonstrated temperatures changes of similar magnitude. Similarly our findings are consistent with other studies where the creation of a humified CO_2_ atmosphere in the surgical wound cavity increased the total wound temperature by 0.5 °C [6]. Whilst such changes may appear insignificant, mild hypothermia has been associated with surgical-wound infection [22, 23], adverse effects on the coagulation system [24], platelet dysfunction [25], increased increases blood loss and allogeneic transfusion requirements [26], and increase adverse cardiac events [27], all of which can be fatal in the context of OLT. Given the long duration and complexity of liver transplantation surgery as well as a high rate of post-operative complications in this patient group, small differences in temperature may be clinically important. Due to the relatively small sample size, differences in clinical outcomes were not observed, however the magnitude of temperature changes observed are consistent with clinical benefits seen in other studies [4].

“Normal” core temperature in healthy adults range between 36.5 °C and 37.5 °C” according to the National Institute for Health and Clinical Excellence guidelines [28]. It is interesting to note that despite intense pre-operative warming strategies employed in both groups in our study, baseline core temperatures at the start fulfilled the definition of “mild” hypothermia in both groups of patients. Reasons for this include exposure of the patient during the insertion of invasive lines, the use of cold antiseptic solution for the line insertions and impaired thermoregulatory function associated with both hepatic failure and general anaesthesia. As expected, we did not observe any significant differences in core temperature at Baseline and at Stage 1 + 60 min, due to the lag time required for the Humigard system to increase core temperature. Similarly, immediately post reperfusion, there were no significant differences in core temperatures measured by the NPP or the PAC. Changes in core temperature immediately post reperfusion depend on the size and temperature of the donor liver, donor liver warm and cold ischaemic times, and temperature and volume of crystalloid solution used to flush the donor liver prior to reperfusion. The difference in temperature change between the two groups at reperfusion was striking. The change of temperature from prior to reperfusion (Stage 3 − 5 min) to post-perfusion (Stage 3 + 5 min) differed by approximately 0.7 °C between the two groups completely reversing the improvement in core temperature seen in the Humigard group up to this point. This anomaly not only explains why we failed to show any significant difference in temperature at Stage 3 + 5 min but also explains the lack of statistically significant difference between the groups at Stage 3 + 60 min, given that 1 h is insufficient to warm a patient by 1 °C. That said, at the Stage 3 + 60 min timepoint, temperatures were noticeably higher in the Humigard group compared to the Control group, and this accelerated warming continued to skin closure, reinforcing the efficacy of the Humigard device in maintaining thermoregulatory homeostasis during OLT.

This study has several methodological strengths. Measurements of core temperatures were all objective variables not amenable to ascertainment bias or manipulation, and our findings were further strengthened by measurements of temperature from two validated physiological temperature-monitoring devices. Selection bias was minimized by randomization and blinding. Comprehensive electronic data collection using electronic temperature monitoring validated the accuracy of our results further increasing the validity of the findings. There are several limitations to our study. First, the sample size was small and the study was powered to demonstrate changes in core temperature, and not changes in adverse outcomes associated with hypothermia. The Humigard system has many other potential benefits, which were not evaluated in the present study. The humidification of the wound prevents dessication of the exposed tissues [29], which in turn may prevent ileus and assist tissue recovery. In addition, given that CO_2_ is bacteriostatic, a reduced rate of surgical site infections might be expected [30]. Pure CO_2_ significantly decreased the growth rate of Staphylococcus aureus at body temperature [31]. Its bacteriostatic effect may explain the low infection rates in patients who undergo laparoscopic procedures. Frey and colleagues also showed that the exposed wound edges were significantly warmer with the Humigard system [9]. This may be associated with improved blood flow and improved healing. These benefits require further clinical research and larger clinical trials before such findings can be validated in the setting of OLT. Finally, the use of any surgical suction apparatus within the abdominal cavity may theoretically deplete the humidified CO_2_ layer. We were not able to measure the impact that the surgical suction had on temperature homeostasis. However, with a humidified CO_2_ flow rate of 10 L/min, any CO_2_ losses through surgical suction would be replaced within seconds. As the surgical suction is not used continuously, we think that it had a negligible effect on the warming capacity of Humigard system.

## Conclusion

In summary, the use of the Fisher & Paykel Humigard® system with standard warming strategies was superior to standardized multimodal strategies in maintaining normothermia in patients undergoing OLT. Use of the device was feasible and did not interfere with any aspects of the surgery. A larger study is needed to investigate if the improved thermoregulation observed is associated with improved patient outcomes.

## Abbreviations

°C: Degrees celsius
APACHE: Acute physiology and chronic health evaluation
CO_2_: Carbon dioxide
HREC: Health research ethics committee
IQR: Interquartile range
MELD: Model for end-stage liver disease
NPP: Nasopharyngeal temperature probe
OLT: Orthotopic liver transplantation
PAC: Pulmonary artery catheter
PaCO_2_: Partial pressure of carbon dioxide
SOFA: Sepsis-related organ failure assessment
